# Early, biomarker-guided steroid dosing in COVID-19 Pneumonia: a pilot randomized controlled trial

**DOI:** 10.1186/s13054-021-03873-2

**Published:** 2022-01-04

**Authors:** Yewande E. Odeyemi, Sarah J. Chalmers, Erin F. Barreto, Jacob C. Jentzer, Ognjen Gajic, Hemang Yadav

**Affiliations:** 1 Division of Pulmonary and Critical Care Medicine, Mayo Clinic, 200 First Street SW, Rochester, MN 55905 USA; 2 Department of Pharmacy, Mayo Clinic, Rochester, MN 55905 USA; 3 Department of Cardiovascular Medicine, Mayo Clinic, Rochester, MN 55905 USA

**Keywords:** C-reactive protein, Corticosteroids, Coronavirus disease 2019

## Abstract

ClinicalTrials.gov identifier (NCT number): NCT03852537, Registered February 25, 2019.

Although corticosteroid administration has been associated with improved outcomes in severe COVID-19 pneumonia, their ideal use remains undefined with a “one size fits all” approach used, irrespective of the individual inflammatory response [1]. Recent studies have highlighted distinct COVID-19 inflammatory phenotypes with differential responses to corticosteroids [2]. Our goal was to assess the feasibility and safety of an individualized, biomarker-guided corticosteroid dosing approach utilizing C-reactive protein (CRP) in patients with pneumonia and acute hypoxemic respiratory failure (AHRF). With the COVID-19 outbreak, a separate COVID-19 trial arm was created, which we report here.

This was a single-center, pilot randomized controlled trial conducted in Mayo Clinic, Rochester, Minnesota from March 2020 through November 2020. Patients with COVID-19 pneumonia and AHRF were randomized to biomarker-guided corticosteroid dosing versus usual care. In the intervention arm, corticosteroid dosing and duration was adjusted to daily CRP level. The dosing algorithm was extrapolated from prior retrospective data [3]. Corticosteroid use and dosing in the usual care arm was determined by the treating physician. Of note, there was a practice change related to corticosteroid administration during the enrollment period following the publication of the RECOVERY trial [4]. All patients had CRP and Troponin measurements on the day of enrollment and then daily for 5 days. The primary outcome was the feasibility of the trial protocol. Secondary outcomes included cumulative corticosteroid exposure, hospital-free days, oxygen-free days, and evidence of cardiac injury (troponin elevation, echocardiographic evidence of new cardiac dysfunction).

Of 41 patients enrolled, 19 were randomized to the intervention arm and 22 to the usual care arm. No significant differences were observed between groups with regards to age, sex, comorbidities, and oxygen delivery devices (see Table 1). Study treatment protocol was followed in 18 (95%) patients in the intervention arm. In the intention to treat analysis the intervention arm had more oxygen-free days (23.5 (21, 25) versus 21 (17, 25), *p* = 0.033) and hospital-free days (21 (18, 22) versus 18.5 (15, 21), *p* = 0.05) than the usual care arm. Daily distribution of CRP in both arms revealed significantly lower CRP levels on day 3 in the intervention arm compared to the usual care arm (see Fig. 1).

**Table 1:** Baseline patient demographics and clinical characteristics

Characteristic	Usual Care (N = 22)	Intervention (N = 19)
Sex, *n* (%)		
Female	9 (41%)	8 (42%)
Male	13 (59%)	11 (58%)
Age (years), median (Q1, Q3)	60.0 (50.0, 66.0)	59.0 (51.0, 81.0)
Race, *n* (%)		
Asian	2 (9%)	0 (0%)
Black or African American	0 (0%)	2 (10.5%)
Unknown/Not Reported	3 (14%)	2 (10.5%)
White	17 (77%)	15 (79%)
BMI (kg/m^2^), median (Q1, Q3)	32.3 (28.5, 39.1)	30.8 (27.2, 39.9)
Saturation/FIO2 Ratio	332 (267.4, 430.5)	339 (250, 423)
Oxygen delivery at randomization, *n* (%)		
HFNC	7 (32%)	3 (16%)
Mechanical ventilation	1 (4%)	1 (5%)
Nasal Cannula	14 (64%)	11 (58%)
Room air	0 (0%)	4 (21%)
COPD, *n* (%)	0 (0%)	0 (0%)
Admitted to ICU at randomization, *n* (%)	10 (45%)	4 (21%)
Sepsis, *n* (%)	0 (0%)	0 (0%)
Diabetes, *n* (%)	5 (23%)	4 (21%)
Asthma, *n* (%)	4 (18%)	1 (5%)
Home oxygen use, *n* (%)	0 (0%)	0 (0%)
Dementia, *n* (%)	0 (0%)	1 (5%)

**Fig. 1:**
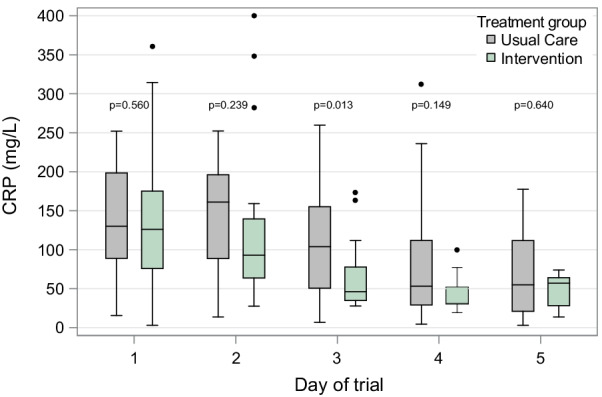
Daily C-reactive protein distribution by arm. CRP: C-reactive Protein

Seventeen (90%) patients in the intervention arm received corticosteroids and 2 patients (due to low CRP levels) did not base on the CRP guided protocol. 11 (50%) patients in the usual care arm received corticosteroids. Steroid use was rare prior to the publication of RECOVERY trial in June 2020. After June 2020, patients in the usual care arm typically received fixed-dose dexamethasone.

When the analysis was restricted to patients that received steroids in both groups, the intervention arm (n = 17) had less cumulative steroid exposure [median 122 (102.0, 160.0.) versus 256 (128, 320) mg, *p* = 0.005], more oxygen-free days [23 (20, 25) versus 17 (8, 22), *p* = 0.032] and no difference in hospital-free days [21 (18, 22) versus 17 (7, 21), *p* = 0.06] than the usual care arm (n = 11).

The results of this single-center pilot randomized controlled clinical trial show that an individualized biomarker-guided corticosteroid dosing approach in pneumonia using CRP is feasible and safe with high adherence to the study protocol. Although not powered to detect differences in patient-centered outcomes, the individualized CRP-guided corticosteroid dosing approach was associated with increased oxygen-free days, hospital-free days, and a lower corticosteroid cumulative exposure in the intervention arm. This is the first study evaluating an individualized biomarker-guided strategy to inform corticosteroid dosing in COVID-19 pneumonia. The protocol outlined can provide a more precise strategy of adjunct drug delivery than the current one-size-fits-all approach. A larger, multicenter clinical trial is needed to determine the efficacy and safety of this approach.

## Data Availability

The datasets generated and/or analyzed during the current study are available on ClinicalTrials.gov identifier (NCT number): NCT03852537 https://clinicaltrials.gov/show/NCT03852537

## Abbreviations

COVID-19: Coronavirus 2019
CRP: C-reactive protein
